# SA-YOLOv11s: A Slicing-Attention YOLOv11s with U-IoU for Oil Leakage Detection in Power Equipment

**DOI:** 10.3390/s26103255

**Published:** 2026-05-20

**Authors:** Daoyuan Liu, Chenlei Liu, Zhijuan Wang, Shiji Zhang, Yulong Yang, Tong Zhao, Xiaolong Wang

**Affiliations:** 1 School of Electrical Engineering, Shandong University, Jinan 250100, China; 202434748@mail.sdu.edu.cn (D.L.);; 2 College of New Energy, China University of Petroleum (East China), Qingdao 266580, China

**Keywords:** insulating oil leakage detection, YOLOv11s, SimAMWS attention mechanism, U-IoU loss function, small object detection, complex background

## Abstract

**Highlights:**

**What are the main findings?**

- An enhanced YOLOv11s-based model is developed for accurate oil leakage detection in power equipment.
- A SimAMWS attention module and U-IoU loss with dynamic scaling are introduced, significantly improving feature extraction and localization accuracy.

**What are the implications of the main findings?**

- The proposed method achieves 97.7% mAP@0.5 and 96.4 FPS, outperforming mainstream detection models in both accuracy and real-time performance.
- It provides reliable and efficient support for intelligent maintenance and monitoring of power equipment.

**Abstract:**

To address the challenges of low detection accuracy and high missed detection rates in insulating oil leakage detection for power equipment—arising from small and densely distributed oil stains, structural occlusion, and complex background interference—this paper proposes a detection method based on an enhanced YOLOv11s (You Only Look Once version 11 small) architecture. First, a dedicated dataset is constructed, encompassing four representative scenarios—small object detection, complex background, multi-object detection and equipment occlusion—to evaluate detection performance. Second, in terms of network design, a proposed attention module, SimAMWS (Simple Attention Module With Slicing), is introduced. This module enhances the model’s sensitivity to subtle and irregular oil stains by utilizing slicing operations and localized energy-based weighting. For bounding box regression, a U-IoU (Unified Intersection over Union) loss is adopted, which incorporates a dynamic scaling mechanism during training to enable the model to focus more effectively on high-quality candidate boxes—leading to improved localization accuracy, particularly suited to the characteristics of oil leakage. Finally, comparative experiments are conducted against mainstream object detectors including SSD (Single Shot MultiBox Detector), Faster R-CNN (Region-based Convolutional Neural Network), YOLOv5s, YOLOv8s, and the baseline YOLOv11s. The proposed method achieves an mAP@0.5 (mean Average Precision at IoU = 0.5) of 97.7% and an mAP@0.5:0.95 of 66.9%, with an inference speed of 96.4 FPS. These results demonstrate that the proposed model delivers higher detection accuracy while maintaining high inference efficiency, making it well-suited for real-time oil leak detection in power equipment and supporting the development of intelligent operation and maintenance systems in the power industry.

## 1. Introduction

With the rapid development of power systems and the continuous growth in load demand, the operational reliability of power equipment has become a crucial factor affecting the safe and stable operation of the power grid [1]. In modern power infrastructure, key components such as transformers and current transformers extensively use insulating oil for electrical insulation and thermal dissipation. However, during long-term operation, issues such as seal degradation, structural fatigue, and external corrosion can result in oil leakage over the surface of the equipment. Such leakage not only compromises the insulation performance but also introduces significant safety hazards, including short circuits, electrical arcing, and even equipment failure with fire. In addition, oil spills may lead to soil pollution, causing serious environmental repercussions [2,3,4]. Therefore, the prompt and accurate detection of oil leakage in power equipment is essential to ensure the safe and reliable operation of the power system.

Currently, the detection of insulating oil leakage in power equipment primarily relies on three types of methods: manual inspection, sensor-based approaches, and image recognition techniques. Manual inspection entails visually examining the external surfaces of electrical equipment and surrounding areas for oil stains as indicators of leakage. However, this method is characterized by low efficiency, a high likelihood of missed or false detections, and limited accuracy in identifying subtle or irregular oil stain targets [5,6]. Sensor-based methods utilize a variety of devices, such as ultrasonic, infrared, fiber-optic, and gas-sensitive sensors, to monitor parameters like acoustic signals, temperature, gas composition, and pressure within the insulating oil in real time. These measurements are then analyzed to assess potential leakage events. For example, Qian et al. employed ultrasonic sensors to capture acoustic signals, which were used to evaluate the condition of transformer insulating oil and infer leakage indirectly [7]. Similarly, Hashim et al. applied humidity and acoustic pattern sensors for detecting leaks in pipelines [8]. Despite their theoretical advantages, sensor-based techniques are often susceptible to environmental fluctuations, which can degrade their performance and limit reliability in real-world applications.

With the widespread deployment of inspection robots and video surveillance systems, large volumes of images can now be collected without relying on manual inspections [9,10]. Image-based analysis enables the early detection of oil leakage, thereby contributing to the safe operation of power equipment. For instance, Xia et al. proposed an oil leakage detection method based on ultraviolet-induced hyperspectral imaging, combining fluorescence–reflectance spectral features with principal component analysis to identify and track various types of insulating oil leakage [11]. Similarly, Lu et al. utilized the fluorescence emitted by insulating oil under ultraviolet illumination, applying the relationship between saturation and brightness in color space to detect leakage in images [12]. However, these methods still largely depend on manual image interpretation. Prolonged visual analysis may lead to operator fatigue, ultimately compromising detection accuracy and efficiency.

With rapid advancements in computer vision, deep learning-based image recognition techniques have emerged as practical methods to enhance detection precision and visualization capabilities, offering new possibilities for intelligent oil leakage identification in power systems. Object detection methods in this domain are generally classified into two paradigms: two-stage detectors, such as Faster R-CNN (Faster Region-based Convolutional Neural Network), which separate region proposal and classification to achieve high accuracy [13]; and one-stage detectors, such as YOLO (You Only Look Once) and SSD (Single Shot MultiBox Detector), which unify localization and classification in a single end-to-end architecture for real-time performance [14,15,16,17]. For example, Yang et al. enhanced the Faster R-CNN model using a ResNet-101 backbone to detect oil leakage defects in power equipment [2,18]. Ji et al. introduced a model based on the PFDAL-DETR (Progressive Feature Decoupling and Alignment Learning with DETR) framework, integrating DETR’s end-to-end detection capabilities with decoupling and alignment modules to improve performance under structurally complex scenarios [19,20,21]. Although these models achieve high detection accuracy, their high computational complexity may increase inference latency and reduce their practicality in real-time power equipment inspection. In recent years, YOLO-based one-stage detection algorithms have gained popularity in practical applications due to their fast inference speed, low deployment cost, and strong real-time capabilities. Progressive improvements in versions such as YOLOv5, YOLOv8, and YOLOv11 have led to architectural refinements and performance enhancements—especially for small object detection [22,23,24,25]. Nonetheless, despite their strong performance in general object detection tasks, YOLO models face difficulties in detecting small-scale oil stains and maintaining robustness under complex visual conditions. Moreover, although YOLO architectures are relatively lightweight, further optimization is still required to reduce computational cost while maintaining detection accuracy. To address these issues, Luo et al. proposed LA-YOLOv8s, a lightweight model tailored for insulating oil leakage detection. This model redesigns the backbone and neck structures to significantly reduce parameters and computational overhead while maintaining detection performance [26]. However, the dataset used in the study was relatively limited in scope, lacking comprehensive coverage of key scenarios such as equipment occlusion, complex backgrounds, small object detection, and multi-target detection. As a result, the model’s generalizability in real-world settings remains insufficiently validated. Given these limitations, improving the detection precision of lightweight models, particularly for small scale and multiple leakage targets in visually complex environments, has become a central focus of current research efforts. In contrast, YOLOv11s demonstrates significant advantages in terms of model compactness, lightweight design, and high inference speed, making it suitable for real-time monitoring applications in power systems, where low-latency image processing is required. Based on the above review, Table 1 summarizes the main categories of existing oil leakage detection methods.

YOLOv11s is selected as the baseline model because it provides a favorable balance between detection accuracy and computational efficiency. Its one-stage detection framework enables fast end-to-end inference, which is important for real-time inspection in power equipment monitoring scenarios. In addition, the C3k2, SPPF, and C2PSA modules in YOLOv11s contribute to efficient feature extraction, multi-scale perception, and attention-guided representation learning. These characteristics make YOLOv11s suitable for detecting small, irregular, and low-contrast oil leakage targets in complex power equipment environments, while leaving sufficient room for further improvement through lightweight attention and localization optimization.

This study presents an improved method for the intelligent detection of oil leakage in power equipment, based on an enhanced YOLOv11s architecture. The proposed enhancements incorporate two key technical innovations. First, the original SimAM (Simple Attention Module) [27,28,29] is extended by introducing a channel slicing strategy and localized normalized energy modeling, resulting in the development of SimAMWS (Simple Attention Module With Slicing). This module is embedded within the YOLOv11s backbone. By generating independent spatial attention maps for each channel, this content-aware attention mechanism enables fine-grained focus on different image regions, thereby improving the model’s ability to extract features from small targets. This enhancement is designed to address common challenges such as reduced detection accuracy in scenarios involving complex backgrounds or equipment occlusion. Second, the U-IoU (Unified Intersection over Union) loss function [30] is integrated into the bounding box regression module. This loss function dynamically scales the predicted bounding boxes during training, guiding the model to prioritize low-quality boxes for faster convergence in the early training stages, while gradually shifting attention to high-quality boxes for enhanced localization accuracy in later stages. This design improves detection performance, particularly in scenarios involving small-scale and multiple leakage targets. To validate the effectiveness of the proposed approach, a dedicated image dataset was constructed, covering four typical operational conditions: equipment occlusion, complex background interference, small object instances, and multi-target scenarios. The dataset simulates the visual disturbances and target variability encountered in real-world power equipment environments, providing a solid foundation for model training and evaluation. Its development enhances the practical applicability of the proposed method and supports future application in power equipment inspection systems. To address these problems, this paper proposes SA-YOLOv11s, whose core idea is to improve local feature perception and bounding box localization within a lightweight detection framework. Specifically, a slicing-based attention mechanism is introduced to enhance the representation of small and irregular oil leakage regions, and a U-IoU loss is adopted to dynamically optimize bounding box regression. These designs enable the model to improve detection accuracy in complex backgrounds, occlusion, and multi-target scenarios while preserving real-time performance.

The main contributions are as follows:

1. A lightweight oil leakage detection model, SA-YOLOv11s, is proposed for power equipment inspection.

2. A SimAMWS slicing-attention module is designed to enhance local feature responses for small and irregular oil leakage targets.

3. A U-IoU loss function is introduced to improve bounding box regression and localization accuracy.

4. A power equipment oil leakage dataset is constructed, and extensive experiments verify the effectiveness and real-time performance of the proposed method.

## 2. Methodology

### 2.1. Overview of the YOLOv11s Model

**Figure 1:**
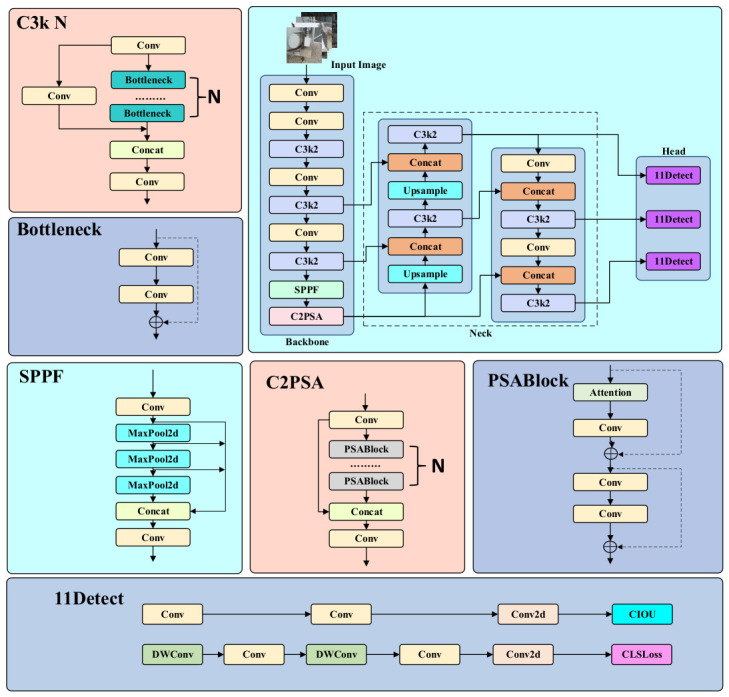
Architecture of the YOLOv11s model.

YOLOv11s represents a recent advancement in the YOLO (You Only Look Once) series, building upon the strengths of earlier versions such as YOLOv5 and YOLOv8. YOLOv11s follows the one-stage detection framework and introduces several lightweight modules for feature extraction and multi-scale representation. Notably, YOLOv11s incorporates the C3k2 (Cross Stage Partial with kernel size 2) module, the SPPF module (Spatial Pyramid Pooling—Fast), and the C2PSA (Convolutional block with Parallel Spatial Attention) module. Each of these components enhances the model’s capability for multi-scale feature extraction and attention-guided learning. The overall architecture of YOLOv11s is illustrated in Figure 1.

The input image is first processed by the Backbone, where it passes through a sequence of Conv (Convolutional layers) and C3k2 modules. These layers progressively reduce spatial resolution while extracting high-level semantic features. Next, the SPPF (Spatial Pyramid Pooling—Fast) module performs multi-scale pooling operations to enhance the model’s capacity to perceive objects at different scales. The C2PSA module introduces a spatial attention mechanism that directs the network’s focus toward salient regions in the image, thereby improving recognition of weak, irregular, or low-contrast targets. The extracted features are then transferred to the Head, where they undergo multi-scale upsampling, concatenation, and further convolutional operations. This process fuses spatial detail with contextual information, enabling joint modeling of object boundaries and semantic features. Finally, the fused feature maps are passed to the Detect module. Through three prediction branches, collectively referred to as 11Detect, the model outputs bounding box coordinates, category labels, and confidence scores, allowing for precise localization and classification of objects across a range of sizes, positions, and shapes. Despite its satisfactory performance in general object detection tasks, YOLOv11s faces limitations when applied to the detection of small-scale oil leakage spots. The continuous downsampling and convolution operations in the Backbone tend to compress spatial detail, which hinders accurate localization of small and irregular leakage traces. In the context of power equipment, such traces are typically small, irregular in shape, low in contrast, and susceptible to interference from complex backgrounds—all of which pose significant challenges to detection accuracy. Furthermore, practical factors such as variable camera angles and equipment occlusion introduce a considerable number of low-quality samples into real-world datasets. YOLOv11s employs the Complete IoU (CIoU) loss function, which imposes strong penalties on these low-quality predictions, potentially impairing the model’s generalization capability. Given these challenges [31], the standard YOLOv11s architecture falls short of meeting the precision and robustness requirements of real-world engineering applications. To address these limitations, this study introduces targeted improvements to both the attention mechanism and the loss function, aiming to enhance the model’s perceptual sensitivity and localization accuracy for oil leakage targets.

### 2.2. Improved Methodology

#### 2.2.1. SimAMWS Module

**Figure 2:**
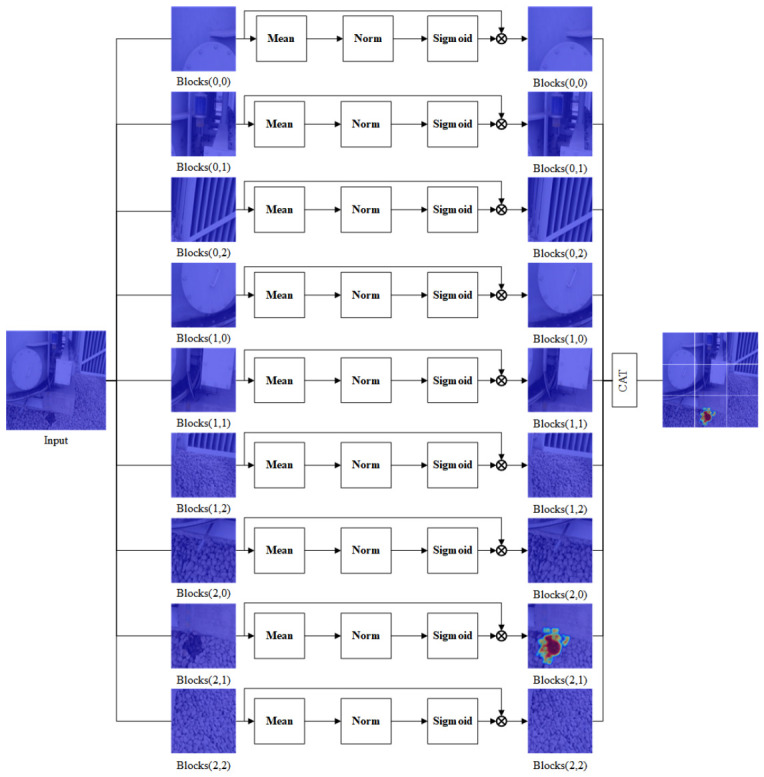
Schematic diagram of the SimAMWS module.

To enhance the model’s feature extraction capabilities under challenging conditions, such as complex backgrounds, small-scale targets, and occlusion, this study introduces the SimAMWS module, an improved version of the original SimAM architecture [27]. The proposed module incorporates a spatial slicing mechanism, wherein the input feature map is divided into multiple sub-regions. Attention weighting is then independently applied within each sub-block, significantly enhancing the network’s ability to capture and model localized information. A schematic diagram illustrating the structure of the SimAMWS module is presented in Figure 2.

For each specific spatial position within a slice, the SimAMWS module employs the following energy function to model the importance of individual features:

(1) $$e_{i,s}^{*}=\frac{4({\hat{\sigma }}_{s}^{2}+\lambda )}{{\left(t-{\hat{\mu }}_{s}\right)}^{2}+2{\hat{\sigma }}_{s}^{2}+2\lambda }$$

In the equation, $e_{i,s}^{*}$ denotes the energy value of a specific position during the processing of input features; and t represents the activation value of a single neuron at that position within a given channel of the feature map. The term λ is a regularization coefficient introduced to prevent instability during training caused by small denominators, with a default value of 0.0001. ${\hat{\mu }}_{s}$ and ${\hat{\sigma }}_{s}^{2}$ denote the mean and variance, respectively, of all other neurons within the same channel, and are defined as follows:

(2) $${\hat{\mu }}_{s}=\frac{1}{M_{s}}\sum_{i\in s}x_{i}$$

(3) $${\hat{\sigma }}_{s}^{2}=\frac{1}{M_{s}}\sum_{i\in s}{\left(x_{i}-{\hat{\mu }}_{s}\right)}^{2}$$

In the equation, *x*_i_ represents the feature value of a specific neuron, and *M*_s_ denotes the number of neurons within the s-th slice. The final attention weight is obtained by normalizing the inverse of the energy value through the sigmoid function:

(4) $${\tilde{X}}_{s}=\mathrm{sigmoid}\left(e_{i,s}^{*}\right)⊙X_{s}$$

(5) $$\mathrm{sigmoid}(x)=\frac{1}{1+e^{-x}}$$

Equations (1)–(4) illustrate the derivation of the energy function. From this derivation, it can be observed that the smaller the value of $e_{i,s}^{*}$, the more linearly separable the target neuron is from other neurons within the same channel, indicating that the corresponding feature is more important for target extraction [32]. In the original SimAM module, both the mean and variance are computed from the entire feature map, which may lead the model to overemphasize background or large-area features. To address this issue, the SimAMWS module introduces a slicing mechanism that divides the feature map into multiple local sub-blocks, where energy weighting is computed independently within each region. This strategy not only enhances the saliency of small targets but also offers favorable properties for parallel computing. Compared with the original SimAM module, SimAMWS reduces part of the additional cost introduced by attention calculation. However, it still introduces a small overhead compared with the baseline YOLOv11s because feature slicing and recombination are required [33,34].

SimAMWS can be regarded as a localized version of SimAM. It calculates the SimAM energy function within sliced local regions rather than over the whole feature map, which reduces background interference and improves the response to small oil leakage targets. Unlike conventional spatial-wise attention, SimAMWS does not generate a global spatial attention map. It also differs from patch-wise self-attention and non-local blocks because it does not model pairwise token relationships or introduce additional learnable attention parameters [35,36,37].

#### 2.2.2. U-IoU Loss Function

IoU (Intersection over Union) is a widely adopted evaluation metric in object detection tasks, used to quantify the spatial overlap between a predicted bounding box and the corresponding ground truth box. It measures prediction accuracy by computing the ratio of the intersection area to the union area of the two boxes. Formally, the IoU is defined as:

(6) $$B_{p}=(x_{p},y_{p},w_{p},h_{p})$$

(7) $$B_{g}=(x_{g},y_{g},w_{g},h_{g})$$

(8) $$IoU(B_{p},B_{g})=\frac{\left|B_{p}\cap B_{g}\right|}{\left|B_{p}\cup B_{g}\right|}$$

In the equation, *x_p_* and *y_p_* denote the center coordinates of the predicted bounding box, while *x_g_* and *y_g_* represent the center coordinates of the ground truth box. *w_p_* and *h_p_* indicate the width and height of the predicted box, respectively, and *w_g_* and *h_g_* denote the width and height of the ground truth box. $\left|B_{p}\cap B_{g}\right|$ refers to the intersection area between the predicted box *B_p_* and the ground truth box *B_g_*, while $\left|B_{p}\cup B_{g}\right|$ denotes the union area of the two bounding boxes.

Conventional IoU and its derivative loss functions—such as Generalized IoU (GIoU) [38], Distance IoU (DIoU), and Complete IoU (CIoU) [31]—primarily rely on static geometric error computations. These methods do not dynamically distinguish between predicted bounding boxes of varying quality, often leading to slow convergence during the early stages of training and insufficient optimization of high-quality predictions in the later stages. This limitation is particularly evident in scenarios involving small objects or densely packed targets, where detection accuracy is often constrained. To overcome these shortcomings, the U-IoU loss function introduces a dynamic scaling mechanism for predicted bounding boxes [30]. This approach balances rapid convergence for low-quality predictions with precise optimization for high-quality boxes. During the box shrinking operation, the IoU value decreases, resulting in a higher calculated box loss. This effectively increases the training weight of high-quality predicted boxes. Conversely, during the box expansion operation, the IoU value increases, leading to a lower box loss, thereby increasing the relative weight of low-quality predicted boxes. This dynamic adjustment mechanism accelerates convergence during training by adaptively emphasizing predictions of varying quality.

The loss function under this method is formulated as follows:

(9) $$loss_{\mathrm{UIoU}}=1-IoU(B_{p}^{\prime },B_{g}^{\prime })$$

(10) $$B_{p}^{\prime }=(x_{p},y_{p},\alpha (t)w_{p},\alpha (t)h_{p})$$

(11) $$B_{g}^{\prime }=(x_{g},y_{g},\alpha (t)w_{g},\alpha (t)h_{g})$$

where *loss*_UIoU_ denotes the bounding box regression loss. In the equation, α(t) denotes the dynamic scaling factor that varies with the training epoch *t.* This factor is designed to increase during the early stages of training—thereby enlarging the bounding boxes—and to decrease during the later stages to gradually shrink the boxes. This dynamic adjustment ensures both smoothness and differentiability in the transition process. In this study, the scaling factor α(t) is defined as

(12) $$\alpha (t)=1+0.5\cos\left(\pi \frac{t}{T}\right)$$

In the equation, *t* denotes the current training iteration, and *T* represents the total number of iterations. This function assigns a relatively large scaling factor to the bounding boxes during the early stages of training, which helps increase the weight of low-quality predicted boxes. As training progresses, α(t) is a time-dependent scaling factor used to adjust the size of the predicted bounding box during loss calculation. Specifically, t denotes the current training iteration and T represents the total number of training iterations. According to this cosine function, α(t) decreases smoothly from 1.5 at the beginning of training to 0.5 at the end of training. When α(t) > 1, the predicted bounding box is enlarged with its center unchanged; when α(t) = 1, the box keeps its original size; when α(t) < 1, the predicted bounding box is contracted.

The physical meaning of this “first expanding and then contracting” process is to adapt the optimization focus of bounding box regression at different training stages. In the early stage, the model usually produces low-quality predicted boxes with inaccurate positions and scales. Enlarging the predicted boxes increases the probability of overlap with the ground-truth boxes, alleviates excessive penalties caused by poor initial localization, and provides smoother gradients for stable convergence. This is particularly useful for small and weak oil leakage targets, whose boundaries are often ambiguous and easily disturbed by complex backgrounds.

As training progresses, α(t) gradually decreases and the scaled boxes change from expansion to contraction. In the later stage, contracting the predicted boxes imposes stricter requirements on the spatial alignment between predicted and ground-truth boxes. Only predictions that are close to the actual leakage region can maintain a high overlap, while boxes with inaccurate boundaries receive larger regression penalties. Therefore, the contraction process encourages the model to refine the boundary location and suppress redundant background regions. In this way, α(t) enables a coarse-to-fine optimization strategy: the early expansion improves robustness and convergence, whereas the later contraction enhances localization precision.

**Figure 3:**
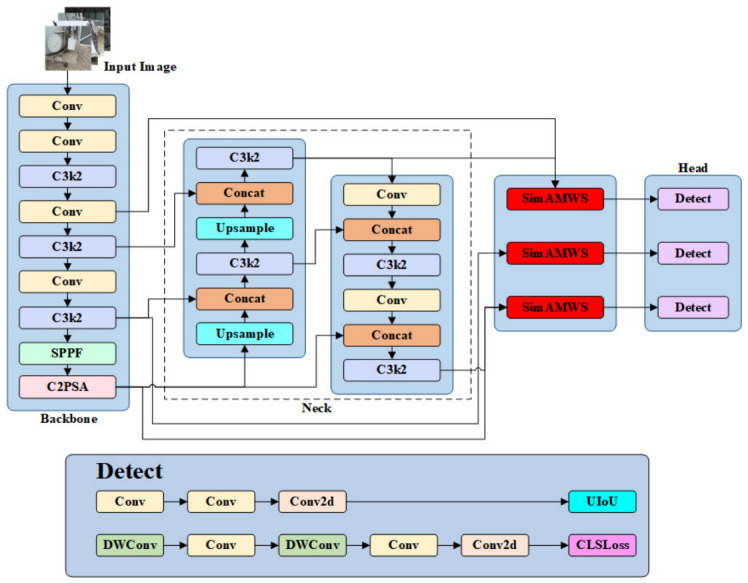
Architecture of the improved YOLOv11s model.

Building on the characteristics of the U-IoU loss function and the SimAMWS attention module, this study further enhances the detection accuracy of insulating oil leakage in power equipment by introducing targeted architectural improvements to YOLOv11s. Specifically, the SimAMWS module is embedded into the Head stage of the network to reinforce spatial attention, while the U-IoU loss function is employed to optimize the bounding box regression process. The overall architecture of the improved YOLOv11s model is illustrated in Figure 3.

#### 2.2.3. Comparison with Related Techniques

To clarify the differences between the proposed modules and related methods, Table 2 compares representative attention mechanisms and bounding box regression losses in terms of their merits and limitations.

Table 2 shows that SimAMWS differs from common attention mechanisms because it does not introduce learnable attention parameters or pairwise spatial relation modeling. Instead, it localizes the SimAM calculation within sliced regions to improve the response to small oil leakage targets. For bounding box regression, U-IoU differs from EIoU, WIoU, and SIoU by introducing dynamic box scaling during training. This provides a coarse-to-fine regression process, although further comparison with other IoU losses on the constructed dataset is still needed.

## 3. Dataset and Experimental Setup

### 3.1. Dataset Construction

To evaluate the performance of the improved YOLOv11s model in the task of insulating oil leakage detection for power equipment, a dedicated oil leakage detection dataset was constructed based on real-world application scenarios. The dataset encompasses a variety of complex environmental conditions, including equipment occlusion, cluttered backgrounds, small object instances, and the coexistence of multiple targets. These representative scenarios are designed to simulate the detection challenges encountered in actual operational environments.

**Figure 4:**
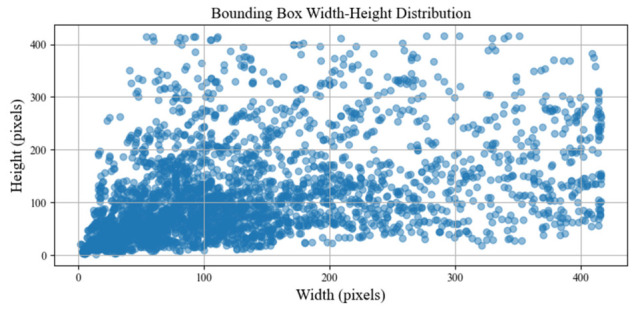
Width–height distribution of bounding boxes in the dataset.

The constructed dataset includes four typical working conditions: (1) small object detection, (2) complex background, (3) multi-object detection, and (4) equipment occlusion. For each condition, 500 images were collected and annotated, resulting in a total of 2000 images. All images were resized to a resolution of 416 × 416 pixels. Annotation was performed using the LabelImg tool, and the dataset was randomly divided into training, validation, and test sets with a ratio of 8:1:1. This tailored dataset serves as a dedicated benchmark for insulating oil leakage detection in power equipment and is designed to reflect the complexities and challenges of real-world deployment scenarios. The width–height distribution of the bounding boxes in the dataset is shown in Figure 4. 

Such a distribution provides a strong foundation for evaluating detection performance across multiple scales and supports further research on intelligent detection of insulating oil leakage [43,44].

The dataset was mainly collected from real inspection images of power equipment and supplemented with laboratory-constructed leakage scenes. Specifically, 1800 images were obtained from real inspection scenarios, and 200 images were collected under controlled laboratory settings. The real inspection images were collected from five substations or inspection sites, covering several types of oil-filled power equipment, including transformers, current transformers, bushings, radiators, conservators, valves, and flange joints.

Image acquisition was conducted using industrial cameras under both substation and laboratory environments. The real inspection images were collected mainly under sunny weather conditions in both real substation scenes and controlled laboratory scenes. Laboratory images were mainly used to supplement rare leakage patterns, small oil stains, and occlusion cases that are difficult to collect in large quantities from operating substations.

### 3.2. Performance Evaluation Metrics

To evaluate the effectiveness of the improved YOLOv11s model in detecting insulating oil leakage in power equipment, this study assesses model performance from two critical dimensions: detection accuracy and real-time capability. Specifically, recall (R), precision (P_e_), average precision (AP), and inference speed (FPS, frames per second) are employed as the primary evaluation metrics. The Intersection over Union (IoU) thresholds are set to 0.5 and 0.5:0.95, corresponding to the computation of mAP@0.5 and mAP@0.5:0.95, respectively. The partial formula for calculating mAP@0.5:0.95 is given as follows:

(13) $$R=\frac{TP}{TP+FN}$$

(14) $$P_{re}=\frac{TP}{TP+FP}$$

(15) $$AP=\int_{0}^{1}P(R)dR$$

(16) $$FPS=\frac{N}{T}$$

In the equation: *TP* (True Positive) denotes the number of correctly detected ground truth targets; *FP* (False Positive) represents the number of incorrect detections; *FN* (False Negative) refers to the number of missed detections; *AP* (Average Precision) is defined as the area under the precision–recall curve across different recall levels; N is the total number of inference images; and *T* denotes the total inference time in seconds.

### 3.3. Experimental Environment and Parameter Settings

To ensure both training efficiency and model performance, all experiments involving the improved YOLOv11s architecture were conducted in a high-performance deep learning environment. The hardware and software configurations are detailed as follows: the experimental platform was equipped with an NVIDIA GeForce RTX 4060 GPU (8 GB VRAM), 16 GB of system RAM, and ran on Ubuntu 20.04. The deep learning framework used was PyTorch 1.13, with CUDA version 11.7 and Python version 3.9. A summary of the experimental hyperparameters is provided in Table 3.

To further validate the superiority of the proposed model, several representative object detection models were selected as baseline comparisons. To ensure fairness and reproducibility, all models—including YOLOv5s, YOLOv8s, YOLOv11s, and the proposed improved version—were trained under identical hyperparameter settings. Specifically, the input image size was uniformly set to 416 × 416 pixels, the batch size was fixed at 8, the initial learning rate was set to 0.001, the optimizer used was stochastic gradient descent (SGD), and the total number of training epochs was set to 150.

SSD features a streamlined architecture and fast inference speed, making it well-suited for real-time detection scenarios. In contrast, Faster R-CNN achieves higher detection accuracy through region proposal extraction and feature alignment, particularly excelling in the recognition of medium-sized targets [45,46,47]. These two models represent distinct paradigms in object detection and serve as strong baseline comparisons for performance evaluation.

## 4. Experimental Results and Analysis

### 4.1. Sensitivity Analysis of SimAMWS Segmentation Configuration

To justify the choice of the 3 × 3 segmentation strategy in SimAMWS, a sensitivity analysis was conducted using different slicing configurations. In this experiment, only the segmentation configuration of SimAMWS was changed, while the network structure, training parameters, dataset split, and evaluation settings were kept the same. U-IoU was not included in this comparison, so that the influence of the slicing strategy could be evaluated independently. For an input image of 416 × 416 pixels, the 2 × 2, 3 × 3, and 4 × 4 configurations correspond to local regions of approximately 208.0 × 208.0, 138.7 × 138.7, and 104.0 × 104.0 pixels, respectively. The 2 × 2 setting preserves more spatial context, but each region still contains a relatively large amount of background information. The 4 × 4 setting provides finer local regions, but it may fragment small and irregular oil leakage areas and introduce additional computational cost. Therefore, the 3 × 3 setting was evaluated as a moderate configuration between local feature enhancement and spatial context preservation.

Table 4 shows that the 3 × 3 configuration provides the best overall performance. The 2 × 2 setting has lower accuracy and FPS than 3 × 3. This is mainly because each sub-region in 2 × 2 is relatively large and still contains considerable background information, which weakens the local attention response to small oil leakage regions. The 4 × 4 setting achieves the highest FPS, but its accuracy decreases because overly fine segmentation may fragment small and irregular leakage targets and reduce the surrounding context needed for discrimination. Therefore, 3 × 3 was adopted as a balanced configuration between local feature enhancement, spatial context preservation, localization capability, and computational efficiency.

### 4.2. Computational Cost and Comparison with Lightweight Attention Modules

To further evaluate the computational cost of the proposed SimAMWS module, GPU memory consumption and inference latency were measured on the same NVIDIA RTX 4060 platform. All models were tested with an input size of 416 × 416 under the same inference settings. Since the purpose of this comparison is to analyze the attention module itself, only YOLOv11s, YOLOv11s + SimAM, and YOLOv11s + SimAMWS are compared. The results are shown in Table 5.

As shown in Table 5, YOLOv11s has the lowest GPU memory consumption and latency. This is expected because it does not include an additional attention module. The original SimAM increases memory consumption to 746 MB and latency to 11.56 ms, indicating a higher computational burden. In contrast, SimAMWS reduces memory consumption to 681 MB and latency to 10.50 ms compared with SimAM. These results show that SimAMWS is more efficient than the original SimAM module. However, it still introduces a small additional cost compared with the baseline YOLOv11s due to feature-map slicing, regional attention calculation, and feature recombination. Therefore, SimAMWS provides a better accuracy–efficiency trade-off than SimAM, rather than completely eliminating the computational overhead of attention calculation.

### 4.3. Comparison with Mainstream Models

To comprehensively assess the proposed model’s computational efficiency and structural complexity, a comparative analysis was conducted against mainstream YOLO-based object detection models, including YOLOv5s, YOLOv8s, and YOLOv11s. The comparison focused on three key aspects: the number of parameters, the network depth (i.e., the number of layers), and the number of floating-point operations (GFLOPs) required for forward inference [48].

The number of parameters refers to the total count of all learnable weights within the model. The number of layers represents the complete sequence of computational operations from input to output, primarily including convolutional layers, pooling layers, and fully connected layers. GFLOPs (Giga Floating Point Operations) quantify the total number of floating-point operations—specifically multiply–accumulate operations—required to process a single image, expressed in billions (10^9^) of FLOPs. These metrics collectively reflect the model’s level of architectural efficiency and computational overhead, providing a quantitative basis for evaluating its suitability for real-world deployment. All measurements were obtained using the torchsummary tool within the PyTorch framework. The results are summarized in Table 6.

As shown in Table 6, YOLOv11s achieves a reduction in both the number of convolutional kernels and the overall channel width by incorporating lightweight modules in place of conventional convolutional structures, combined with an optimized feature fusion strategy. In the proposed model, although both the SimAMWS attention mechanism and the U-IoU loss function are integrated—each being simple and parameter-free—only a slight increase in network depth is observed, and the GFLOPs rises marginally to 21.8. This indicates that the architectural enhancements do not introduce model redundancy. Compared to YOLOv8s, which has a significantly higher parameter count, the proposed model reduces GFLOPs by more than 24%, demonstrating superior computational efficiency and greater suitability for real-time inspection in real-world applications.

**Figure 5:**
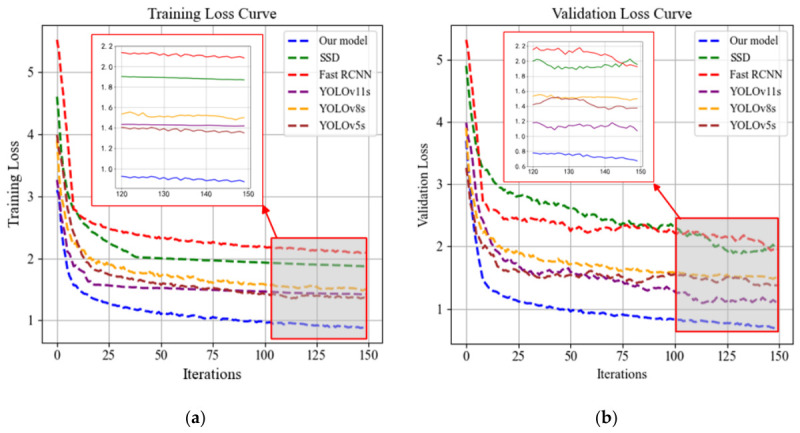
Loss curves of different detection models on training and validation sets. (**a**) Training loss curves. (**b**) Validation loss curves.

To evaluate the convergence behavior and training stability of the proposed model, this study compares the loss function trends of several mainstream object detection algorithms during both the training and validation phases. The selected models include the classical SSD and Faster R-CNN, as well as the more recent and high-performing YOLOv5s, YOLOv8s, YOLOv11s, and the proposed improved model. Figure 5 illustrates the variation in loss values on the training and validation sets as a function of the number of training iterations for each model.

Figure 5a and b respectively illustrate the loss function trends for each model on the training and validation sets. Overall, all models show a sharp decline in loss during the first 30 epochs, followed by gradual stabilization—indicating successful initial convergence. However, a more detailed comparison reveals that the proposed model achieves a significant reduction in loss within the first 10 epochs, demonstrating a notably faster convergence rate than the other models. This efficiency can be attributed to the dynamic weighting mechanism of the U-IoU loss function, which prioritizes the optimization of low-quality bounding boxes in the early training stages and progressively shifts focus toward high-quality boxes in later stages. This strategy enables more efficient gradient utilization and supports a gradually refined learning objective. Clear differences are also observed in the final steady-state loss values across models. The proposed model achieves a training loss stabilized around 0.55 and a validation loss near 0.58, both outperforming the baselines. In contrast, YOLOv5s and SSD stabilize at considerably higher values—approximately 0.9/1.0 and 2.0/2.2, respectively—demonstrating the superior convergence behavior and generalization capability of the proposed approach. Further analysis of the training phase between epochs 120 and 150 reveals that the amplitude of loss fluctuations reflects the model’s gradient stability post-convergence. Smaller fluctuations indicate smoother parameter updates and more complete convergence. Among all the compared models, the proposed model exhibits the smallest fluctuation amplitude, indicating superior training stability.

To further validate the overall performance advantages of the proposed model in the task of insulating oil leakage detection, a comparative evaluation was conducted against several mainstream object detection models, including SSD, Faster R-CNN, YOLOv5s, YOLOv8s, and YOLOv11s. The comparison was based on five key metrics: precision (Pre), recall (R), mean Average Precision at IoU threshold 0.5 (mAP@0.5), mean Average Precision across IoU thresholds from 0.5 to 0.95 (mAP@0.5:0.95), and frames per second (FPS). The detailed results are presented in Table 7.

**Figure 6:**
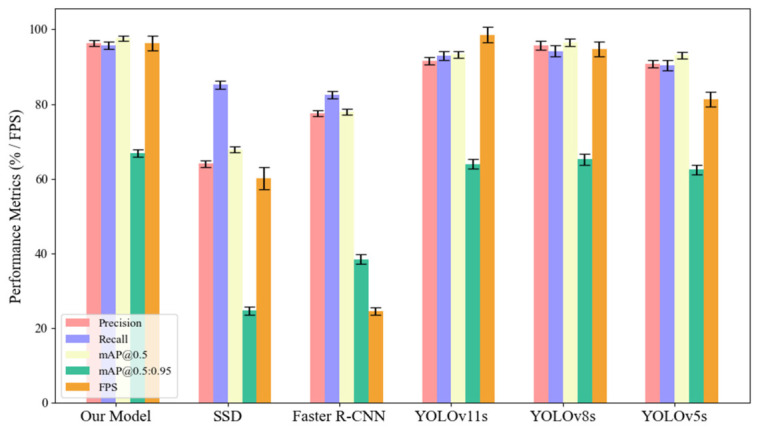
Bar chart comparison of detection metrics across different models.

**Figure 7:**
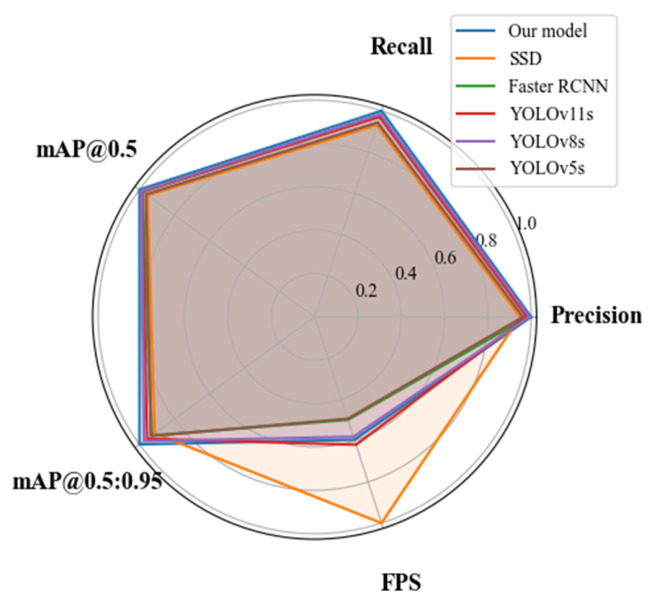
Radar chart comparison of detection metrics across different models.

As shown in Table 7, the proposed model achieves the best performance across all accuracy-related metrics. Specifically, it reaches an mAP@0.5 of 97.7% and an mAP@0.5:0.95 of 66.9%, representing improvements of 1.4% and 2.9%, respectively, over the baseline YOLOv11s model. Moreover, despite the integration of an attention mechanism and a refined loss function, the model achieves an inference speed of 96.4 FPS—surpassing both YOLOv5s and YOLOv8s—which demonstrates its strong capability for real-time detection. Overall, the results confirm that the proposed enhancements improve detection accuracy while maintaining real-time performance and training stability, making the model suitable for deployment in power equipment inspection tasks. To further evaluate the model’s robustness, five independent training experiments were conducted. The corresponding results are presented in Figure 6 and Figure 7.

As shown in the figures, the proposed model not only achieves the highest scores across four accuracy-related metrics—precision, recall, mAP@0.5, and mAP@0.5:0.95—but also exhibits significantly shorter error bars compared to the baseline models. This indicates that the model maintains stable performance under varying training conditions. In contrast, although SSD and Faster R-CNN demonstrate certain advantages in recall and FPS, they exhibit noticeably larger error ranges, reflecting limited robustness. Specifically, for the mAP@0.5:0.95 metric, the proposed model maintains an error margin within ±1.0%, whereas Faster R-CNN and YOLOv5s exceed ±1.5%, indicating that the proposed method achieves more accurate and consistent target localization. Furthermore, despite incorporating an attention mechanism and an optimized loss function, the proposed model sustains a high inference speed of 96.4 FPS, with variability confined to ±2.0 FPS. This demonstrates that performance gains were achieved without significantly compromising inference efficiency. Through multiple experimental repetitions and comprehensive error analysis, it is evident that the proposed model consistently outperforms other methods across all evaluation metrics, making it suitable for complex power equipment inspection tasks that demand both high accuracy and high reliability.

### 4.4. Ablation Study

To evaluate the individual contributions of the SimAMWS attention module and the U-IoU bounding box loss function to the overall detection performance, a series of systematic ablation experiments were conducted based on the YOLOv11s backbone. These experiments compared different combinations of the two components, and the resulting performance was analyzed across five key evaluation metrics. The detailed results are summarized in Table 8.

Table 8 reports the ablation results of the proposed modules. When only U-IoU is introduced, Precision, Recall, mAP@0.5, and mAP@0.5:0.95 increase from 91.6%, 93.0%, 93.3%, and 64.0% to 93.0%, 94.0%, 94.7%, and 65.4%, respectively. Since the network architecture is unchanged, this improvement is mainly attributed to better bounding box regression during training. The FPS remains close to that of the baseline, which is expected because U-IoU is not involved in the inference stage. The standard SimAM module improves mAP@0.5 to 95.2%, but the FPS drops to 86.5. This indicates that its attention calculation brings additional computational cost. In comparison, SimAMWS achieves 96.8% mAP@0.5 and 65.5% mAP@0.5:0.95 while maintaining 95.2 FPS. The result suggests that the slicing strategy improves local feature representation for leakage regions with a smaller impact on inference speed. The best performance is obtained when SimAMWS and U-IoU are used together. The final SA-YOLOv11s model achieves 96.4% Precision, 95.8% Recall, 97.7% mAP@0.5, and 66.9% mAP@0.5:0.95. Compared with the SimAMWS-only model, mAP@0.5 and mAP@0.5:0.95 are further improved by 0.9 and 1.4 percentage points, respectively. The slight FPS difference between SimAMWS and SimAMWS + U-IoU is within the normal fluctuation of inference-time measurement, rather than a change caused by the loss function. Overall, compared with the baseline YOLOv11s, SA-YOLOv11s improves mAP@0.5 by 4.4 percentage points and mAP@0.5:0.95 by 2.9 percentage points, with only a 2.2 FPS decrease. This indicates a practical trade-off between detection accuracy and real-time performance.

To reduce the influence of a single train-validation-test split, a repeated random split experiment was conducted for SA-YOLOv11s. The dataset was randomly divided five times using the same 8:1:1 ratio, and the model was trained and evaluated under identical parameter settings. Since the purpose of this experiment is to verify the stability of the reported performance of the proposed model, only SA-YOLOv11s was evaluated in this repeated-split setting. The repeated random split results for SA-YOLOv11s are summarized in Table 9. 

### 4.5. Detection Result Visualization

**Figure 8:**
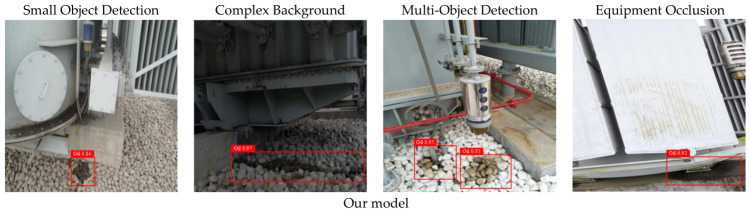

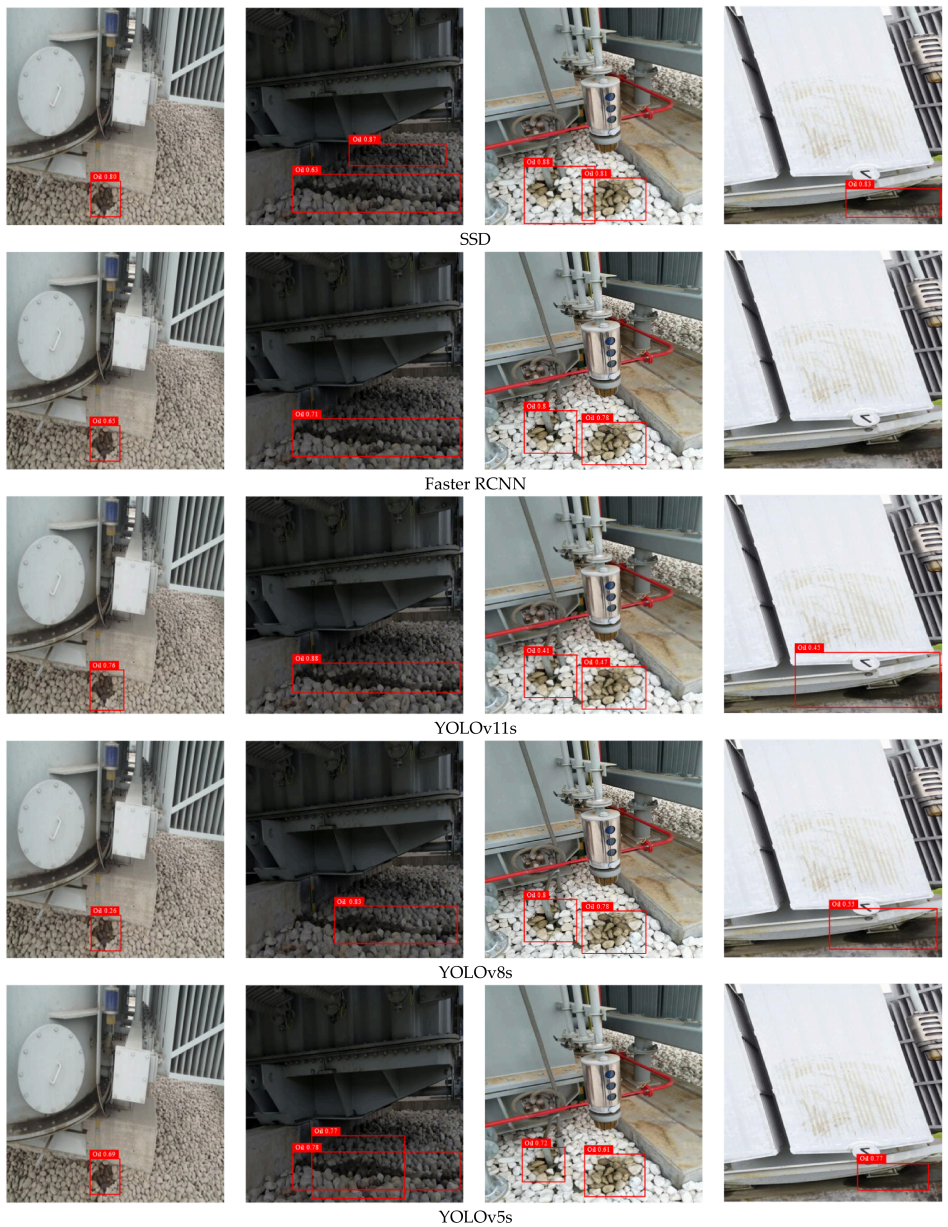
Visual comparison of oil leakage detection results under typical working conditions across different models.

To further validate the practicality and robustness of the proposed model under diverse and complex conditions, four representative working scenarios for insulating oil leakage detection in power equipment were selected for visual comparison experiments. These include: (1) small object detection, (2) complex background, (3) multi-object detection, and (4) equipment occlusion. These scenarios closely reflect the typical challenges encountered in actual substation environments, making them both representative and realistic. To comprehensively assess the model’s generalization ability, five mainstream object detection models were selected as baselines. Visual comparisons were conducted on the same set of images, contrasting their detection outputs with those of the proposed model. The results are presented in Figure 8. 

In the visualizations, the confidence score reflects the model’s certainty in its predictions—higher values indicate greater confidence that the predicted region contains a true target. As observed, the proposed model performs particularly well in small object detection tasks. Even for extremely small leakage regions located at the image corners—occupying less than 2% of the total image area—the model is able to accurately localize the target with a high confidence score of 0.86, whereas other models either assign low confidence scores or fail to detect the target altogether. In scenarios involving complex background interference, such as stone-paved grounds where the color and texture closely resemble those of oil stains, most models are susceptible to background noise, leading to false positives or significant drops in confidence. In contrast, the proposed model maintains a high confidence level of 0.97, demonstrating strong robustness against background complexity.

In multi-target detection scenarios, where multiple leakage regions are densely distributed and some targets are located in close proximity, conventional models such as SSD tend to generate overlapping bounding boxes, leading to reduced detection clarity. In contrast, the proposed model consistently detects all targets with clear separation and maintains an average confidence score above 0.93, demonstrating strong capability in handling densely packed scenes. In equipment occlusion scenarios—where portions of the leakage areas are obscured by structural components or affected by strong reflective surfaces—Faster R-CNN fails to detect certain targets, while YOLOv11s exhibits noticeable misalignment of bounding boxes. The proposed model, however, accurately localizes even partially occluded leakage regions, with confidence scores consistently around 0.92, underscoring its robustness in complex industrial visual environments.

Comprehensive analysis indicates that the proposed model achieves accurate detection of small, densely distributed, and partially occluded targets across all four representative scenarios, consistently outperforming mainstream object detection algorithms. These results validate the effectiveness of the proposed improvements in enhancing detection precision under complex and challenging conditions. To more intuitively demonstrate the feature attention capabilities of different models under various detection scenarios, this study introduces Grad-CAM visualization for comparative analysis [49]. The resulting attention heatmaps reveal how each model responds to representative scenes, as shown in the figure. The selected scenarios are consistent with the previous visualizations and cover four typical conditions: small objects, complex backgrounds, multiple targets, and equipment occlusion. The comparison includes the proposed improved model alongside five mainstream object detection models: SSD, Faster R-CNN, YOLOv11s, YOLOv8s, and YOLOv5s.

**Figure 9:**
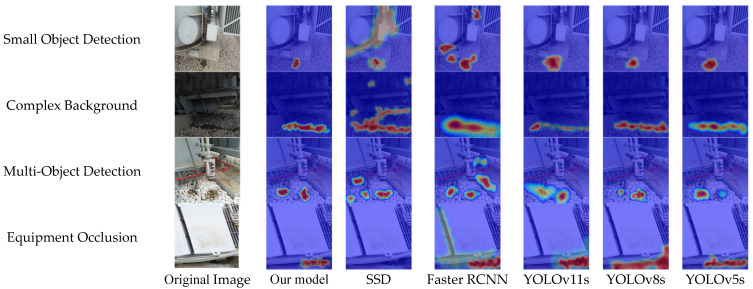
Grad-CAM heatmap visualization comparison of different models under typical detection scenarios.

As shown in Figure 9, SA-YOLOv11s produces more concentrated activation regions around oil leakage areas than the comparison models. In small-target and complex-background scenes, the heatmaps of SSD and YOLOv5s are more dispersed, while the proposed model shows stronger responses near the annotated leakage regions. In multi-target scenes, SA-YOLOv11s separates adjacent leakage regions more clearly. In occlusion scenes, the activation regions are mainly distributed around the visible parts of the leakage area. These results suggest that SimAMWS helps the network focus on leakage-related local features under challenging visual conditions. Nevertheless, missed detections and false detections may still occur in some difficult scenes. For example, extremely small or heavily occluded oil stains may not be recognized, and low-contrast leakage regions may only be partially detected. In addition, rust, dust, shadows, reflections, or water-like marks may be confused with oil leakage. These cases show that SA-YOLOv11s still needs to be improved under complex field conditions.

## 5. Conclusions

This study proposes SA-YOLOv11s for insulating oil leakage detection in power equipment. The method is designed to address common detection difficulties in field images, including small leakage regions, dense oil stains, equipment occlusion, and complex background interference. Based on YOLOv11s, two targeted improvements are introduced: the SimAMWS attention module and the U-IoU loss function.

(1) The main contribution of SimAMWS is to improve local feature representation. By dividing the feature map into local regions and calculating attention responses within each region, SimAMWS helps the model focus on small and irregular leakage areas while reducing the influence of large background regions. Compared with the original SimAM module, the slicing strategy reduces part of the attention-related computational cost.

(2) U-IoU further improves the bounding box regression process. Its dynamic scaling strategy provides a coarse-to-fine optimization mechanism during training. In the early stage, the expanded predicted boxes improve the tolerance of low-quality predictions and help stabilize training. In the later stage, the contracted boxes impose stricter localization constraints, encouraging more accurate boundary alignment. This is useful for oil leakage targets with small size, irregular shape, and partial occlusion.

(3) Experimental results show that SA-YOLOv11s achieves 97.7% mAP@0.5 and 66.9% mAP@0.5:0.95, outperforming the baseline YOLOv11s and other compared detection models. The model contains 9.46 million parameters and requires 21.8 GFLOPs, while maintaining an inference speed of 96.4 FPS. Although the FPS is slightly lower than that of the original YOLOv11s, the decrease is small compared with the improvement in detection accuracy. These results indicate that the proposed method provides a practical balance between accuracy and real-time performance for power equipment inspection.

(4) The visualization results and Grad-CAM heatmaps show that SA-YOLOv11s produces more concentrated responses around leakage-related regions under small-target, complex-background, and occlusion conditions. This supports the effectiveness of combining local attention enhancement with improved bounding box regression.

Several limitations should be noted.

(1) This study focuses only on insulating oil leakage in power equipment. Other liquids, such as rainwater, condensed water, cleaning liquid, or lubricant stains, were not used as independent detection categories. Therefore, the proposed model should be regarded as an oil leakage detector rather than a general liquid-stain classifier. In real inspection scenes, visually similar regions such as water marks, rust, dust, shadows, and reflections may still lead to false detections.

(2) The dataset size is still limited compared with large-scale object detection datasets. Although the repeated random split experiment reduces the influence of a single train-test split, it cannot fully replace validation on larger independent field datasets. Moreover, the proposed model has not yet been fully evaluated on external datasets or completely unseen substations. Future work will collect more independent field samples from additional substations, equipment types, weather conditions, and imaging devices to evaluate the real-world generalization ability of the proposed model.

## Figures and Tables

**Table 1:** Summary of existing oil leakage detection methods.

Parameter Name	Merits	Limitations
Manual inspection	Simple and direct	Low efficiency; subjective
Sensor-based methods	Real-time signal monitoring	Sensitive to environment
Image-based methods	Provide visual evidence	Small targets remain challenging

**Table 2:** Comparison of related methods.

Module	Merits	Limitations
Spatial attention	Highlights key regions	Global maps may weaken small targets
ECA [39]	Lightweight	Limited local spatial modeling
Coordinate Attention	Preserves position information	Adds coordinate encoding operations
Non-local block	Captures long-range dependency	High memory and computation cost
SimAM	No learnable parameters	Global calculation may be affected by background
SimAMWS	Enhances small-target response	Requires predefined slicing
EIoU [40]	Improves geometric regression	No dynamic scaling during training
WIoU [41]	Adjusts sample contribution	Sensitive to focusing strategy
SIoU [42]	Improves geometric alignment	Adds extra geometric terms
U-IoU	Coarse-to-fine regression	Further comparison on this dataset is needed

**Table 3:** Hyperparameter settings for the improved YOLOv11s model.

Parameter Name	Value
Input Image Size	416 × 416
Batch Size	8
Initial Learning Rate	0.001
Optimization Algorithm	SGD
Number of Training Epochs	150
Bounding Box Loss Function	U-IoU

**Table 4:** Sensitivity analysis of different segmentation configurations in SimAMWS.

Segmentation	Precision (%)	Recall (%)	mAP@0.5 (%)	mAP@0.5:0.95 (%)	FPS
2 × 2	95.5	94.3	96.5	65.3	93.7
3 × 3	95.8	94.1	96.8	65.5	95.2
4 × 4	94.9	93.7	95.9	64.8	96.6

**Table 5:** Computational cost comparison of different models.

Segmentation	GPU Memory (MB)	Latency (ms)
YOLOv11s	612	10.14
YOLOv11s + SimAM	746	11.56
YOLOv11s + SimAMWS	681	10.50

**Table 6:** Comparison of model parameters, network depth, and computational complexity across object detection models.

Model	Parameters (M)	Number of Layers	GFLOPs
YOLOv5s	7.23	213	16.5
YOLOv8s	11.2	225	28.8
YOLOv11s	9.46	319	21.7
Our model	9.46	322	21.8

**Table 7:** Performance comparison of detection models on the test set.

Model	Precision (%)	Recall (%)	mAP@0.5 (%)	mAP@0.5:0.95 (%)	FPS
Our model	96.4	95.8	97.7	66.9	96.4
SSD	64.1	85.2	67.8	24.7	60.1
Faster-RCNN	77.6	82.5	78.0	38.5	24.6
YOLOv11s	91.6	93.0	93.3	64.0	98.6
YOLOv8s	95.7	94.2	96.6	65.2	94.7
YOLOv5s	90.8	92.8	93.1	62.5	81.3

**Table 8:** Impact of each improvement module on detection performance in the ablation study.

Model	Improved Module	Precision (%)	Recall (%)	mAP@0.5 (%)	mAP@0.5:0.95 (%)	FPS
YOLOv11s	None	91.6	93.0	93.3	64.0	98.6
YOLOv11s	+U-IoU	93.0	94.0	94.7	65.4	98.4
YOLOv11s	+SimAM	93.5	93.6	95.2	64.7	86.5
YOLOv11s	+SimAMWS	95.8	94.1	96.8	65.5	95.2
YOLOv11s	+SimAMWS + U-IoU	96.4	95.8	97.7	66.9	96.4

**Table 9:** Repeated random split results of SA-YOLOv11s.

Run	Precision (%)	Recall (%)	mAP@0.5 (%)	mAP@0.5:0.95 (%)
1	96.4	95.8	97.7	66.9
2	96.1	95.5	97.4	66.5
3	96.6	95.7	97.8	67.0
4	95.9	95.3	97.2	66.2
5	96.3	95.6	97.5	66.6
Mean ± Std	96.3 ± 0.3	95.6 ± 0.2	97.5 ± 0.2	66.6 ± 0.3

## Data Availability

Data available on request due to restrictions of privacy and legal reasons.

## Abbreviations

The following abbreviations are used in this manuscript:

Faster R-CNN	Faster Region-based Convolutional Neural Network
YOLO	You Only Look Once
SSD	Single Shot MultiBox Detector
DETR	DEtection TRansformer,
PFDAL-DETR	Progressive Feature Decoupling and Alignment Learning with DETR
SimAM	Simple Attention Module
SimAMWS	Simple Attention Module With Slicing
U-IoU	Unified Intersection over Union
C3k2	Cross Stage Partial with kernel size 2
SPPF	Spatial Pyramid Pooling—Fast
C2PSA	Convolutional block with Parallel Spatial Attention
Conv	Convolutional layers
CIoU	Complete IoU
R	Recall
P_e_	Precision
AP	Average Precision
FPS	Frames Per Second
TP	True Positive
FP	False Positive
FN	False Negative
SGD	Stochastic Gradient Descent
GFLOPs	Giga Floating Point Operations
